# Behavioral symptoms and caregiver burden in dementia

**DOI:** 10.4103/0019-5545.44905

**Published:** 2009

**Authors:** K. S. Shaji, Roy K. George, Martin J. Prince, K. S. Jacob

**Affiliations:** Department of Psychiatry, Medical College, Thrissur - 680 596, Kerala, India; 1 College of Nursing, M.G.DM Hospital, Kangazha, Kottayam, Kerala, India; 2 Department of Epidemiological Psychiatry, Centre for Public Mental Health, Institute of Psychiatry, King's College, London; 3 Department of Psychiatry, Christian Medical College, Vellore - 632 002, India

**Keywords:** Dementia, caregiver burden, Behavioral symptoms

## Abstract

**Background:**

Dementia care in developing countries will continue to be provided by co-resident caregivers at home. Behavioral and Psychological Symptoms of Dementia (BPSD) are difficult to manage at home. Interventions leading to reduction or remission of reduction or remission of BPSD will be of immense help in the management of these patients.

**Materials and Methods:**

The nature and prevalence of BPSD in a community sample of patients with dementia was assessed by a clinician. The impact of these symptoms on the caregiver was assessed by measures of burden of care and the psychological well being of the caregiver. Another rater carried out these assessments independently.

**Results:**

Prevalence of BPSD was very high and they were more common in patients with Alzheimer's Disease than patients with Vascular Dementia. They were rated as troubling to most caregivers. Caregiver burden was associated with adverse effects on the mental health of the carer.

**Conclusions:**

To be effective, dementia care services in developing countries need to focus on management of BPSD at home. Development of a low cost, effective and sustainable dementia care service should be given due importance by the policy makers in the developing world.

## INTRODUCTION

Dementia is a rapidly growing problem in the developing regions of the world. These societies are characterised by low levels of awareness regarding dementia as a chronic degenerative brain syndrome, and by an absence of supportive health and welfare services. There is heavy reliance upon families as the cornerstone of support and care. Almost all patients with dementia are looked after at home by a co-resident family member. This situation is unlikely to change in the near future, as institutional care is neither affordable nor culturally acceptable.

Despite this, there are no formal evaluations from developing countries of the practical, emotional, and economic impact of caring for a family member with dementia. Dementia, in common with other mental health conditions, exerts a disproportionate effect on family and co-residents. A widely accepted notion within developing country societies of the family as endlessly supportive caregivers may not be true[1] Even where care is exemplary, it is essential that the impact of providing care on the family, and on the wider community be quantified.

In a qualitative study of caregivers of persons with Alzheimer's disease[2] we found that majority of caregivers were young women, often daughters-in-law of patients affected by dementia. The principal sources of caregiver strain were Behavioral problems associated with the dementia syndrome, and incontinence. Strain was exacerbated by the lack of supportive response by local health services, and by lack of support and, sometimes, criticism from other family members. Family conflict was commonly encountered. The majority of caregivers experienced significant deterioration in their mental health.

Behavioral and Psychological Symptoms of Dementia (BPSD) is a term used to describe a heterogeneous range of psychological reactions, psychiatric symptoms, and behaviors occurring in people with dementia of any etiology.[3] It represents an important clinical dimension of dementia that has until recently been ignored from both research and therapeutic points of view. Because of their frequency and their adverse effects on patients and their caregivers, these disturbances should be ascertained and treated in all cases of dementia. Remission or reduction of BPSD is also known to produce remarkable improvement in the functional abilities of the patient. The present study examines the prevalence of BPSD in a community sample of patients with dementia and its impact on the caregivers.

## MATERIALS AND METHODS

The study was conducted at Engandiyur Panchayath of Thrissur District, in Kerala, India. A Panchayath is a rural administrative area under the local administration. It has a total population of about 19000 out of which 1979 are aged 60 years or more. The Thrissur Centre of the 10/66 Dementia Research Group is in the process of developing a community based dementia care service for this population.

Cases of dementia were identified using a novel case identification method which was developed and validated at the Thrissur center of 10/66 Dementia Research Group in India.[4] This method makes use of trained women health workers to identify potential cases of dementia in the community. Clinicians confirmed the clinical diagnosis of DSM-IV[5] dementia, and then rated them on Clinical Dementia Rating Scale.[6] Cases which received a rating of mild or moderate severity criteria only were included in the study. Behavioral symptoms were scored on the caregiver-rated BEHAVE - AD[7] which is a 25- item rating scale with assessments on a four-point severity score. The primary care giver was asked to give details about the symptoms which were present during the one month period prior to interview. A symptom was coded as present only when it was reported as present during this specified period.

Subjects of this study took part in two other studies conducted by the10/66 Dementia Research Group[8,9]. Assessments for the Caregiver Pilot Study included the12-item General Health Questionnaire (GHQ-12) as a measure of psychiatric morbidity,[10] and the Zarit Burden Interview (ZBI) as a measure of caregiver strain.[11] The ZBI has 22 items that assess the caregiver's appraisal of the impact their involvement has had on their lives. Scores of Zarit Burden Interview and GHQ -12 were used as measures of Caregiver strain in the present study. A score of 3 or more on GHQ -12 was considered as indicative of mental health morbidity in the primary caregiver.

### Analysis

Prevalence and nature of BPSD was estimated using BEHAVE-AD scores. The data was analysed for associations between the scores of BEHAVE-AD and a) patient factors - age, gender, dementia subtype and clinical severity and b) carer outcomes - scores on ZBI and GHQ-12.

Mean and standard deviation were employed to describe continuous variables, while frequency distributions were obtained for categorical data. The chi square test was used to assess the significance of associations for categorical variables. Student's t-test was used to test the associations for continuous data. Pearson's correlation coefficient was used to test the association between continuous variables. The statistical software SPSS for Windows Release 6.1.3 was employed for the analysis.

## RESULTS

The study sample consisted of 29 cases who were assigned DSM IV diagnosis of dementia and were rated as having dementia of mild to moderate severity as per CDR. The mean age of the sample was 78.3. There were 23(79.3%) women in the sample. All of them lived with their families. According to the information available with local health workers, no elderly person from the study area was receiving institutional care at the time of the study. We received good co-operation from the families and all the caregivers agreed to participate in the study.

### Clinical characteristics of people with dementia

Of the 29 patients, eighteen met DSM IV criteria for the diagnosis of Dementia of Alzheimer's type. Four of these cases also met the consensus criteria for Dementia with Lewy Bodies (DLB).[12] Ten cases received the diagnosis of Vascular Dementia and one was diagnosed as dementia due to other general medical conditions as per the DSM 1V. See Table 1 for other characteristics of the sample.

**Table 1:** Characterstics of patients with dementia

Characteristic	No.	%
Diagnosis
Alzheimer's disease	14	43.8
Vascular dementia	10	34.5
Dementia with lewy bodies	4	13.8
Others	1	3.4
Education
Illiterate	15	51.7
A little	12	41.4
Secondary	2	6.9
BEHAVE-AD global rating
No trouble	8	27.6
Mild	8	27.6
Moderate	5	17.2
Severe	8	27.6
Marital status
Married	7	24.1
Widow	22	75.9
Sex
Female	23	79.3
Male	6	20.7

### Nature and prevalence of BPSD

Behavioral disturbances were extremely common in this sample. Twenty-eight patients (96.6%) had one or more of BPSD as assessed by BEHAVE -AD. The total scores varied between 1 and 30 with a mean of 11.4 [Table 2]. The total score on BEHAVE- AD scale correlated inversely with age of the patient. This relationship was statistically significant (Pearson's correlations coefficient -0.3984; *P*=0.032).

**Table 2:** Scores of Zarit burden interview and BEHAVE-AD

Characteristic	Mean	SD
ZBI score	33.8	16.8
BEHAVE-AD total score	11.3	9.2
BEHAVE-AD: Anxiety.	0.6	1.2
BEHAVE-AD: Affective	1.0	1.4
BEHAVE-AD: Hallucinations	1.3	2.1
BEHAVE-AD: Agg ressiveness	2.1	2.4
BEHAVE-AD: Activity disturbances	2.2	2.3
BEHAVE-AD: Delusions	3.3	3.2

Zarit burden interview (ZBI)

Paranoid and delusional ideations along with activity disturbances were the most frequently identified among the seven BEHAVE-AD symptom categories [Table 3]. “People are stealing things” was the most common delusion. Fourteen patients had this particular belief and paranoia was seen in 13 cases. See Table 4 for the content of delusions. Hallucinations were reported to be present in 12 (41.3%) patients. Ten patients had visual hallucinations and nine had auditory hallucinations. One patient had an olfactory hallucination.

**Table 3:** The nature of behavioral and psychological symptoms of dementia

Items on sub-scales of BEHAVE-AD	Prevalence (%)
Paranoid and delusional ideation	19 (65.5)
Hallucinations	12 (41.3)
Activity disturbances	19 (65.5)
Aggressiveness	15 (51.7)
Diurnal rhythm disturbances	13 (44.8)
Affective disturbance	13 (44.8)
Anxietties and phobias	7 (24.1)

**Table 4:** Types of paranoid and delusional ideation

Type of delusions	Number (%)
People are stealing things	14 (48.3)
Home is not one's home	10 (34.5)
Abandonement	5 (17.2)
Infidelity	2 (6.9)
Paranoia	13 (44.8)
Others	7 (24.1)

### Type of dementia and BPSD

Patients with AD and DLB together had significantly higher total scores on BEHAVE-AD than patients with Vascular Dementia [Table 5]. Activity disturbances and Delusional thinking were also more common in this group than the group of patients with Vascular Dementia.

**Table 5:** Comparison of factors associated with specific dementia: Vascular dementia vs other dementias

Factor	Other dementias (n=19)	Vascular dementia (n=10)	Significance
Score of caregiver burden as per ZBI	35.8 sd 16.7	30.0 sd 17.2	ns
BEHAVE-AD total score*	13.9 sd 9.5	6.5 sd 6.5	*P*=0.037
BEHAVE-AD score on activity disturbances	2.8 sd 2.4	0.9 sd 1.4	*P*=0.026
BEHAVE-AD score on delusions	4.4 sd 3.3	1.4 sd 2.0	*P*=0.16
Age of the patient	76.5 sd 9.1	81.7 sd 8.6	ns
Currently married	5 (26%)	2 (20%)	ns
Female gender	14 (74%)	9 (90%)	ns
Moderateand severe carer stress rating on BEHAVE-AD	11 (58%)	2 (20%)	ns
Illiterate	9 (53%)	6 (60%)	ns
Carer GHQ -12 cases	15 (79%)	6 (60%)	ns

* Other comparisons on BEHAVE-AD subscales not significant

### Impact of BPSD on caregivers

The Global Rating of BEHAVE-AD provided an assessment of overall caregiver distress due to BPSD. The symptoms were rated as not at all troubling to the caregiver or dangerous to the patient in eight (27.6%) cases. Out of the remaining 21, BPSD was rated as mildly troubling to eight (27.6%) and moderately troubling to five caregivers. In eight cases, BPSD were rated as severely troublesome and intolerable to the caregivers.

There was a significant association between the total score of BEHAVE-AD and high levels of caregiver distress on global rating. Symptoms like delusional thinking, activity disturbances and aggressiveness were more likely than other symptoms t be rated as troublesome to the caregiver [Table 6]. There was, however no correlation between the BEHAVE-AD scores and scores on Zarit Burden Interview or GHQ caseness. High scores on Zarit Burden Interview were significantly associated with GHQ caseness [Table 7].

**Table 6:** Factors associated with global rating of severity: Comparison of patients with no or mildly troubling symptoms vs patients with moderate or everely troubling symptoms as per BEHAVE-AD

Factor	Group with no or mild symptoms (n=16)	Group with moderate or severe symptoms (n=13)	Significance
Score of Caregiver Burden as per ZBI	30.0 sd 17.8	38.5 sd 14.8	ns
BEHAVE-AD total score*	5.5 sd 7.1	18.5 sd 5.8	*P*=0.000
BEHAVE-AD activity disturbances	0.93 sd 1.4	3.7 sd 2.2	*P*=0.000
BEHAVE-AD delusions	1.6 sd 2.3	5.5 sd 3.0	*P*=0.001
BEHAVE-AD aggression	.5 sd 1.0	4.2 sd 2.0	*P*=0.000
Age of the patient	78.6 sd 8.6	77.9 sd 10.1	ns
Currently married	5 (31%)	2 (15%)	ns
Female gender	12 (75%)	11 (85%)	ns
Illiterate	9 (56%)	6 (46%)	ns
GHQ -12 total score	4.6 sd 2.8	5.0 sd 3.1	ns
GHQ -cases	11 (69%)	10 (779%)	ns
Vascular dementia	8 (50%)	2 (16.%)	ns

* Other comparisons on BEHAVE-AD subscales not significant

**Table 7:** Factors associated with GHQ caseness: Comparison of caregivers with a score of 3 or more on GHQ-12 with the rest of the caregivers

Factor	GHQ cases (n=21)	GHQ non cases (n=8)	Significance
Score of caregiver burden as per ZBI	39.6 sd 4.9	18.6250 sd 11.7	T value 3.57 df 27 *P*=0.001
BEHAVE-AD total score*	11.4 sd 9.8	11.1 sd 8.1	ns
Age of the patient	78.0 sd 8.7	78.9 sd 10.8	ns
Currently married	6 (29%)	1 (13%)	ns
Female gender	15 (71%)	8 (100%)	ns
Moderate and severe carer stress rating on BEHAVE-AD	10 (48%)	3 (38%)	ns
Illiterate	11 (52%)	4 (50%)	ns
Vascular dementia	6 (29%)	4 (50%)	ns

* All comparisons on BEHAVE-AD subscales not significant

## DISCUSSION

This is study reports the nature and prevalence BPSD from a community sample of patients with mild to moderate dementia from a rural India. Earlier studies on BPSD were on clinic based samples[13–15] The high prevalence of BPSD reported here shows that these symptoms are common in developing as in developed country settings. These symptoms are stressful for the co-resident caregivers, who lack support and guidance from the health care delivery system.

### BPSD in developing countries

There is every reason to believe that BPSD is present across cultures in the developed as well as developing regions of the world. But, the prevailing low levels of public awareness about dementia in India and other developing regions of the world[16] have many implications. It reduces the chances of correct identification and management of BPSD in the community. Moreover, the relatives frequently misinterpret these symptoms as deliberate misbehaviour. Others could even misinterpret BPSD as evidence of the poor quality of care provided by the family. Allegations of this kind only add to the misery of the caregiver.

### Psychotic symptoms in dementia

Total scores of BEHAVE-AD as well as the scores on the two subscales (Paranoid and delusional ideation and activity disturbances) were more frequent in patients meeting the criteria for AD and DLB when compared to patients with vascular dementia. It is possible that psychotic symptoms are more common in neuro-degenerative dementias. Psychotic syndromes in dementia need further study. Prospective studies using standard definitions of psychosis[17] may help to clarify associations between behavioral and psychotic symptoms. For example, do delusions and hallucinations drive activity and diurnal rhythm disturbances? A common patho-physiological basis could have important implications for drug and non-drug management strategies. Certain delusions with the content that “People are stealing things” or “Ones house is not one's own home” are commonly seen in patients with dementia. Our experience is that when such delusions are present without obvious activity or diurnal rhythm disturbances, then non-pharmacological interventions may be preferred. However, such clinical observations need to be tested in future studies of BPSD.

### Impact of caring for a person with dementia

Caring is associated with substantial psychological strain as evidenced by high rates of psychiatric morbidity and high levels of caregiver strain. We did not find any association of BEHAVE-AD scores either with caregiver burden or with caseness as per GHQ-12. This might have been due to the small size of the study sample. However, there is evidence from the Global ratings of Behave -AD that the BPSD in general and symptoms like activity disturbances; aggressiveness and delusions in particular are indeed troublesome to the caregiver. Data from our qualitative study also strongly support this view.[2] Many factors might positively and negatively influence the experience of caregiver burden and modulate the psychological impact of providing care to the demented relative. Future studies should look at various factors that might increase or lessen the burden of care. The high rate of GHQ caseness among the caregivers and the strong association between carer burden and GHQ scores highlights the adverse impact of caring on the psychological well being of caregivers.

### Need to identify and manage BPSD

Behavioral and Psychological Symptoms of Dementia (BPSD) are common and cause significant distress to patients and caregivers. These symptoms could be misinterpreted by the relatives especially in developing societies where public awareness of dementia as health condition is very low[2,18]. Providing information about BPSD and educating the family members may be particularly important and effective in these settings. BPSD are generally considered to be more amenable to interventions than the cognitive symptoms of dementia. That is yet another reason to focus on these symptoms when we develop and deliver community based interventions. Given the prospect of increase in the number of old people affected by dementia in the developing world, we need to develop strategies to assist families who care for their demented relatives at home. Development of simple, culturally acceptable, non- pharmacological interventions for the management of BPSD in the community would be a right step in this direction. Once the feasibility and cost effectiveness of such interventions are established, they could form an important ingredient of community based dementia care services in developing countries.

### Limitations of the study

This study has used a case identification method that might have a low sensitivity and thus would have lead to selective exclusion of mild cases of dementia, especially those without prominent Behavioral symptoms. Thus the prevalence rate of BPSD reported here cannot be considered as representative of all cases in the community. Small sample size was yet another limitation. Population based estimates of BPSD are needed for accurate estimates of the prevalence of BPSD in the community. This is will soon be addressed in the population based studies undertaken by the 10/66 Dementia Research Group.
